# Counteracting the Schools’ Demon: Local Social Changes and Their Effects on the Participation of Roma Children in School Education

**DOI:** 10.1007/978-3-030-52588-0_8

**Published:** 2020-07-08

**Authors:** Stefánia Toma

**Affiliations:** 1 Instituto Universitário de Lisboa (ISCTE-IUL), CIES-IUL, Faculty of Architecture, University of Lisbon, Lisboa, Portugal; 2 Open University (UAb), Instituto Universitário de Lisboa (ISCTE-IUL), CIES-IUL, Lisboa, Portugal; 3 Research, Romanian Institute for Research on National Minorities, Cluj-Napoca, Romania; 4 Romanian Institute for Research on National Minorities (RIRNM), Cluj-Napoca, Romania; 5 Babeș-Bolyai University, Sociology Doctoral School, Cluj-Napoca, Romania

**Keywords:** School education, Interventions, Migration, Roma, Romania

## Abstract

The aim of the article (The empirical material leading to the present chapter results from the research effort “*MigRom—The Immigration of Romanian Roma to Western Europe: Causes, effects, and future engagement strategies*”, a project funded by the European Union’s Seventh Framework Programme under the call “Dealing with diversity and cohesion: the case of the Roma in the European Union” (GA319901). I also used the results and experiences of earlier fieldworks starting with 2000 in Bighal (the name of the localities were changed in order to respect the identities of the people) that were financed through Open Society Institute, Visegrad Funds, CERGE-EI through GDN and WIIW, respectively Inclusion 2007 through PHARE 2004. Earlier version of the article was presented at the GLS Conference in Nicosia (Cyprus) in 2017. The article was finalized in the framework of a visiting research programme at TARKI-POLC receiving funding from the European Union’s Horizon 2020 research and innovation programme under grant agreement No. 730998, “*InGRID-2*—*Integrating Research Infrastructure for European expertise on Inclusive Growth from data to policy”*.) is to inquire into the interconnectedness of large number of factors that carry the opportunity and possibility of improving school participation of Roma children in Romania.

I argue that the inherent deficiencies of the educational system, starting with the structural constraints and ending with the psycho-social context in which Roma (or minoritized, marginalized, vulnerable) children learn, can be and are challenged by initiatives, strategies or processes that fall out of the immediate range of the strict framework of the educational system. Bourdieu used the Maxwell’s demon as a metaphor to illustrate the reproduction of socio-economic inequalities in the framework of school system. But this ‘demon’ might be challenged with more or less success if we step out and look for possible ‘tools’ to counteract this demon. Two such cases are presented in this chapter. One is a project implemented with and by the local Roma community using external financing and the other one is the participation of the members of the communities in international migration and use of remittances. I will emphasize that independently of the type and amount of the mobilized resources the individuals and/or communities are able to create and proactively make good use of path-departing opportunities through mechanisms of redefining and changing contextual constraints thus improvements can be observed in the school participation of the Roma children (PS. PS. The article was written before the COVID-19 pandemic hit the world. Its effects seems to neutralize the positive impact of the above mentioned processes: the slow steps taken in improving the socio-economic situation of the Roma seems to be stopped; prejudices and ethnic hatred seems to be stronger; access to services for Roma communities get more difficult, including to education: in this context, a further research question is how on-line schooling changed or will change the participation of Roma children?).

Education—whether formal or informal—permeates all aspects of our lives beginning from our early existence. It is part of a complex system, the elements of which interacts with and mutually influences each other. It not only includes national level politics and policy, it also affects community relationships, and at a micro-level influences the lives of individuals and their families. Thus, almost every stage of life of a family is framed and structured by education and schooling, too. The daily routine of a family with children is deeply graven by the school programme. Education and schooling—implicitly everything that is connected to it—can offer prestige, success, feeling of completion and delusion, it can be the site of equity or injustice, it can design aspirations, can improve social relations, but it can have disruptive effects and can restrict access to different services and domains as well, especially if one does not participate in it out of various reasons.

Not coincidently was the school system of primary importance for Bourdieu, who calls it Maxwell’s demon,^1^ using it as a metaphor for maintaining socio-economic inequality among students: it “maintains the pre-existing order, that is, the gap between pupils endowed with unequal amounts of cultural capital.” (Bourdieu 1998, p. 20).

Ultimately, in some cases, irrespective of the fact that one participates or not, the school system might contribute to creating Catch-22-like situations. This is particularly true for marginalized, vulnerable children, specifically for the Roma children. If they are not enrolled in school or they drop-out earlier as compared to their peer-group, later they have to face the—many times insurmountable—disadvantages brought by having less or no school education. On the other hand, the social situation of Roma children in school is frequently described as lacking the same conditions as their non-Roma peers’, for example: poorer nutrition, higher exposure to health risks, and poorer housing conditions (O’Nions 2007, pp. 146–155), which might otherwise lead to effective and successful engagement in school activities (Payne 2019), it rather emphasize the ethnic gap in education (Papp 2011). Hence the cumulated disadvantages would reinforce their already precarious socio-economic situation.

Bourdieu’s “demon” goes beyond the lack of capital (whether economic, social or cultural). It also encompasses the attitudes of their peers and the teachers that Roma children have to deal with while being in school, thus the concept of equality of opportunities (Maclean 2003) gains more dynamic and complex substrata. Thus, besides the effects of the parent’s background and wealth, the quality of school infrastructure and teaching practices, the lack or inadequacy of educational programs and policies (Walther et al. 2016), Roma children have to face stereotypical and/or prejudicial attitudes and behaviors based on their ethnicity, language and socio-economic position.

Consequently, to accomplish improvements in this complex life-arena might seem a Sisyphean task, however I will argue that it is not impossible. Improvements in the school education of the Roma children can be induced either from the top and/or from below by changing the characteristics of the interdependent factors that influence the way educational process unfolds. The implementation of policy measures and legislation frames the organization and practices of school education contributing to the creation of availability of the objective chances and opportunities (including infrastructures) needed for the success of particular social categories (Bourdieu and Passeron 1977). People’s strategies (including teachers’, parents’ and the children’s) and community-based interventions may come in completion, reinforcement and improvement of the top-induced conditions and might also contribute to changes in participation in the educational system for disadvantaged children (Drown 2019).

Maneuvering, building strategies or even just drifting with the tide in order to make ends meet in this complex context both for children and parents is a demanding process that claims long-term financial, social and emotional investment with no guarantees of success. One out of the many strategies of the families that might influence directly or indirectly the Roma children’s school educational attainment—among other aspects of their everyday life—is engaging in short- or long-term migration, investing remittances in goods or activities that might improve the school participation of their children.

This chapter aims at shedding light on the interconnectedness of factors that might affect the school participation of Roma children, focusing on an individual (i.e migration) and a community level (NGO project) strategy. Despite the fact that these are processes that develop outside the immediate school environment, they can both have a stimulating or thwarting effect on the school participation of Roma children. I will argue from a relational and processual perspective borrowed from Norbert Elias (1978, 1997), that improvements—even if modest—in the school participation of socially and economically deprived children can be reached by mobilizing local community resources independently of the institutional context (legal, infrastructural and human resources) of schools, while the quality of the institution positively supports and consolidates the efforts of the individuals.^2^

In this article I will bring two examples of interventions that have had positive influence on school participation. I will emphasize that independently of the type and amount of the mobilized resources, the individuals and/or communities are able to create and proactively make good use of path-departing opportunities through mechanisms of redefining and changing contextual constraints.

Throughout the chapter, I will provide glimpses of statistical and ethnographic data on the initial context and what changes occurred in two Transylvanian villages over a more than 10 years period, focusing on the local conditions that generated those changes and answering the question of how these changes happened.^3^ While the focus in this chapter is local, it should be highlighted, that during this period significant initiatives and events had happened in Romania, that have also inevitably influenced local level processes. The *Romanian Government’s Strategy for Improving the Condition of Roma 2001-2010* was developed, later it was replaced by the *Strategy of the Government of Romania for the Inclusion of the Romanian Citizens belonging to Roma minority for the period 2012–2020*. Romania also participated in the *Decade of Roma Inclusion 2005–2015*, the *European Platform for Roma Inclusion*, the *EU Framework for National Roma Integration Strategies*, as well as other relevant strategic initiatives that aimed/aims at the social-economic inclusion of Roma minority in the fields of education, health, employment, housing, culture and social infrastructure and designed the direction of the interventions. With the 2007 EU accession of Romania, international travel (labor force migration) of Romanian citizens became easier. However it was immediately followed by the global financial-economic crises that penetrated the job market situation on an unprecedented level, as well as having impacted the local informal employment possibilities. This resulted in the growing number of Roma migrants seeking employment opportunities in Western European countries.

The chapter is structured as follows: first, I will present the case of an NGO initiative that was implemented at the beginning of the 2000s with the contribution of the local Roma community and its influence on the school participation of the Roma children, contrasting it with the situation in another village from Transylvania. Then, I turn to another major moment in the lives of the local communities, that of when Roma started to participate in international migration in both villages and discuss how migration and remittances made its contribution to the improvement of school participation of Roma children.

## Civil Society’s Community-Based Interventions

In the early 2000s during fieldwork in Bighal I woke up early in the morning on the barely distinct noises of some family members whispering and rustling around the kitchen that also served as living room. It was a usual kindergarten and school day for the children. Their father had already left for work, but the girls had to prepare their long hair in tresses, their mother tried to convince them that they have breakfast, at least a modest one, and at the same time check their school timetable, their schoolbag, their clothes, school supplies and maybe a favorite doll to be packed. One yawn came after the other for everybody. Eventually, they left. One of the girls was taken to the state kindergarten in the center of the village; the bigger child was left on the way at the elementary school. The scene is familiar for many who have school-aged children irrespective of their ethnicity.

But hardly can we say that it was typical for all Roma households in Bighal. There were other families who during those years had a different daily program. The early morning haste maybe was a constant for every family, but in the compact community the parents were waiting for the arrival of the educational practitioner to take the children to the recently built Roma community center for the morning hours of preparation, so that later that day they could go, already prepared, to the central state kindergarten that functioned in an old building. In the Roma community center, simply named by locals as “ *Gypsy* *kindergarten*” those days, children were prepared to get used to the activities of the central kindergarten under the supervision of an ethnic Romanian educator who also knew Hungarian language and some Romani.

This community center was a project implemented by an NGO and functioned for 2 years. The project required the active involvement of the local community who contributed by producing clay bricks for the construction of social houses and the community center. At the beginning of this period, in 1999/2000 13 Roma children were enrolled in the kindergarten, out of them 12 were more than 6 years old (out of the 156 enrolled children from the village). While in the elementary school (grades I–IV) there were 16 Roma children enrolled at the Hungarian section and 40 at the Romanian classes. In the secondary school (grades V–VIII) there were 5 Roma children in Hungarian classes and 18 in Romanian classes, but with the majority of them only attending up to the fifth or the sixth grade. In the eighth grade only one Roma child was attending the Hungarian section.

More than a decade later, the oldest child of my host family already graduated university in a nearby town, the middle child is a university student in the same town and the third and youngest member of the family, the boy, is preparing to go to high school. The mornings are more relaxed from educational point of view. During the last few years their father used to work in construction in different countries being the member of an ethnically mixed local work-group. This opportunity greatly contributed to the significant growth of the household income. They were able to afford more school-related expenses parallel with the improvement and extension of the house (building additional room and a bathroom). Meanwhile the community center was transformed offering space for different community activities (public baths for example), depending on the available funding. The so-called Roma “kindergarten” was closed and never re-opened because it was considered as a separated educational space and that its programme overlapped with that of the state kindergarten. The old, small and not properly equipped central kindergarten was moved to a newly built, two-storey building with many facilities, extended programme and more employees. In 2015/2016 there were 65 Roma children enrolled in kindergarten between 3 and 6 years old. There were 88 Roma children in the elementary classes and 31 in the secondary school, and 2 Roma children were told that they should attend school but they dropped out. These years the number of Roma children enrolled in kindergarten and school remained stable at a relatively high level: during the 2013/2014 school year there were 57 Roma children in the kindergarten while 147 children in the I–VIII grades and in 2014/2015 50 children in the kindergarten and 155 in the I–VIII grades.

The most spectacular increase in attendance of Roma children was registered in kindergarten. As already mentioned in 1999/2000 school year 13 Roma children were enrolled in the kindergarten, 15 years later there were 65 Roma children, while there were no major changes in the size of the local Roma community.^4^ Though not at the same pace, improvements were observed in elementary- and secondary school enrolment and graduation as well in this locality (see Table 8.1.). Pre-school attendance is viewed as crucial in ensuring further educational opportunities (Drown 2019) and in reducing not only early school drop-out 5 (Roth and Moisa 2011, p. 518; FRA 2018, p. 11), but also reduces dramatically of being MPI (multidimensional poverty index) poor by 12.9% (Ivanov and Kagin 2014, p. 67).

**Table 8.1:**
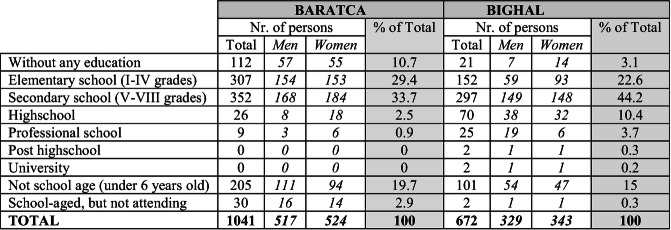
The school participation (the highest level) of Roma persons (men and women) in Bighal and Baratca according to the MigRom survey (2016)

Data source: MigRom project, RIRNM

Table 8.1 shows the highest school level graduated by Roma comparing the two localities shown in the MigRom survey. We can observe that Roma in Bighal spend significantly more years in school than their peers in Baratca.^6^ Although there is room for improvement in both localities as regards school education, there are a number of reasons why Roma in Bighal have a higher level of participation. While there are no significant differences between the socio-economic situation of the Roma households comparing the two localities, the broader social and infrastructural context shows some notable differences. For example, the most obvious one is that access to pre-school education developed differently in Bighal. It was already presented that in Bighal there was a specific program implemented that contained a component focusing on pre-school activities. There is a vocational highschool in Bighal and the distance to the nearest small town is also smaller compared to Baratca. Roma in Bighal speak at least three languages,^7^ and although most of the Roma children are enrolled at the Romanian language section, they also have access to some programs that targets Hungarian-speaking pupils (events, excursions, scholarships, etc). By comparison, in Baratca, the Roma are Romanian speakers, there have been comparably less educational programs implemented, and there is no high school in the locality or in the closer region at all (the closest locality with any kind of highschool and easiest to reach is aprox. 40 km far). This later puts an additional financial burden on families that in the context of severe poverty and marginalization just few households can afford. In addition, ethnic relations on locality level influences school-participation (Kruse and Kroneberg 2019). In Baratca the social distance between Roma and non-Roma is high that inevitably marks social interactions in any context. As a result, ‘ white-flight’ of Hungarian students started to develop, leaving the local school resembling more and more like a Roma only school. On the other hand, in Bighal the interactions between Roma and non-Roma are more frequent and are not heavily loaded with prejudices and distance-keeping attitudes (Toma and Fosztó 2018a).

However this is just a cross-sectional image of the current educational context and we can only draw some already well-established conclusions at this point. The following paragraphs will try to present a more nuanced analysis on the educational situation of Roma children in these two localities highlighting that this present situation displayed in Table 8.1 stemmed from the complex combination and interaction of different factors and contexts that generated transformations in the local society and through this the educational situation as well.

To gain a more accurate image of how participation in schooling has changed over different periods and generations, we grouped the persons based on their year of birth into different categories.^8^ More precisely, we selected the generation of children who were pre-school aged (between 4 and 6 years old) in the period when they had the chance to be part of the program implemented in Bighal and compare their school participation to the previous generations in both villages. We use data from Baratca as well despite the fact that there were no comparable programs implemented at that time in the village, but, to some extent, the data are comparable to the results in Bighal. As Table 8.2 shows, the differences are striking, and not only when comparing the two villages, but when comparing the school participation of different age-cohorts in Bighal as well. The ratio of persons without any education in Baratca among those born between 1994 and 1999 is very high—almost half of that generation skipped schooling, but those who did enroll they stayed more in school compared to the previous generations (more persons graduated some kind of highschool). That was the period of structural rearrangements and political repositioning when it became clear that most of the Roma found themselves as unemployed and hadn’t found viable alternatives yet, integration programs and NGO projects were not present, while the effects of the global economic crises started to make themselves felt, thus the Roma felt into the institutional dead spot for a while. But it is also the period when, in the wake of EU accession, authorities renounced at the school-registration conditionality of the universal child allowance between 2006 and 2009 (Raț et al. 2020), and this period covers more or less the generation of children that ought to begin school during those years (children born between 1994 and 1999). In Bighal the education data denotes a better situation. More than half of the children born between 1994 and 1999 graduated some form of highschool (including vocational schools as well) considerably improving the situation compared to the previous age-cohorts.^9^

**Table 8.2:**
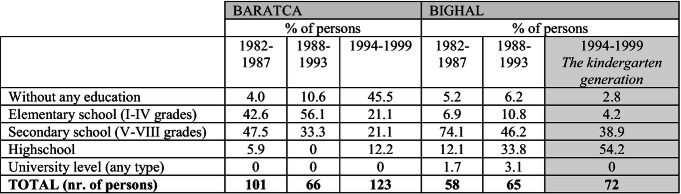
The highest graduated school level of Roma persons born between 1982–1987, 1988–1993 and 1994–1999 comparing Bighal and Baratca (%)

Data source: MigRom project (RIRNM)

These children were of pre-school age at the time of the functioning of the community center in the Roma neighborhood, and even if our data does not permit to identify individually those who participated in the program and those who did not, we can assume that among others there is a generational model-following effect. This is illustrated by qualitative data, as well. The narratives of both Roma and non-Roma about this community project underline the positive effects of it, as the local Roma experts put it at several years distance:

Many don’t go to school after a year or two, because they fail and they can’t continue. Now, it’s better that we did this day-care through the association (the local Roma NGO), that children attend before entering elementary school and they learn how to draw, to handle a pencil and anything they need to know. The educator works nicely with the children, she teaches them Romanian, they receive food, as well. Hope that it will be better from now on. (…) But now people realize that school means something. That those who have school [education] think better, can talk to each other. Not like the savages. School is very important, like in the past – if you had school, you had profession, too. (interview, Roma expert, 2003, Open Society Institute project, Bighal)

[the Gypsy kindergarten]… but that was good from some point of view, because there the children learnt something…they learnt Romanian, because there they used the Romanian ( language), and learnt a bit how to behave, and they learnt…something…how to eat. When they enrolled in the first class, they already knew a bit to write, or what to say…how to handle the pencil, to draw, to count…something, anything. (interview, Roma Health Mediator, 2005, CERGE-EI project, Bighal)

Exactly there were in kindergarten, all my children [the Roma children from the elementary school]. And it counts enormously, very much indeed, that they went there. The socialization has already started, that is very important, and anyway they have a vocabulary already. And they speak Romani at home, they don’t speak Hungarian or Romanian. And [because of this] their vocabulary is poor, reduced [when they enter school].(…) But with this kindergarten is much easier. Because they already have some knowledge, you have something to start with. It is much easier at the kindergarten because you work with games, images…so, it counts a lot for the children if they attend kindergarten. (interview, Romanian elementary school teacher, Romanian section, II. grade, 2005, CERGE-EI project, Bighal)

While in Bighal the externally financed (by an international foundation) and community-based intervention—at least partially (because it was needed for the interplay of other factors as well, as we will see later)—explains the improvements in the school participation of these children, how can we explain the relative improvement in Baratca?

## Evolving Migration Patterns and Impacts

The migration of Roma in increased numbers started later than the migration of their non-Roma co-villagers in both localities due to the declining formal and informal work opportunities after 2007. In both villages approximately 60% of the surveyed households^10^ had at least one member of the family who worked abroad for shorter or longer periods of time (most of them between 3 and 6 months during the summer). Almost all households declared that they spent money received or brought back home from abroad in the year preceding the survey. Most of the spending went into buying consumer goods (food, clothes, home appliances), improving, building or buying houses, spending on health, only a few families (five from Baratca) managed to invest financial remittances into small businesses. But there are significant differences both in the way migration patterns unfolded and in its effects. The migratory patterns of the Roma communities in these villages were shaped by the degree and modes of maintenance of social distance between the Roma and local majority. The hierarchically organized ethnic categories and networks built along these categories shaped the way remittances were spent and invested at home (Toma and Fosztó 2018a). This resulted in either competitive or cooperative relations between ethnic groups, depending on the local symbolic hierarchies that were challenged or reinforced (Toma and Fosztó 2018b, p. 43). The migration and remittance induced changes are visible on local level in both villages, but in Baratca these changes are not always perceived positively by the local non-Roma. While they acknowledged some aspects as beneficial changes (new skills learned, improvements in the lived spaces, changed mentalities, etc.), some locals interpreted the geographical desegregation process of the Roma community (moving in the center of the village) as a threat, as “invasion of the village” and expressed their fear that the Roma eventually will become the numerically dominant population. Local Hungarians interpret these processes as feeling of “loss of own space” that individually was translated into “white flight”-like decisions. Because there are more Roma children in the local school, some of the parents had decided that they rather take their children to the all-Hungarian school in the nearby village.

Another significant difference worth mentioning is that, comparing to Baratca, a considerably higher number of households in Bighal declared that they spent the remittances on the education of their children. This means also that they invested more in the clothing of the children, bought school supplies and were able to contribute more to the constant needs of the school (contributing to the class money, paying for excursions, buying the necessary books and other school supplies). Conway and Cohen (1998, p. 28) underlined that in the case of poor households/communities the importance of consumption expenditure should not be underestimated, as it has developmental effect on long term, because better food, more clothes or any other consumable contributes to the overall improvement in the households’ general well-being (for example, health). Similarly, investment in health and education should be defined as productive investment. So far, we have seen that in Bighal more households spent remittances directly on education, than in Baratca. That does not mean that in Baratca the spending of remittances does not have a share in the improvement of continuation of schooling. As Bloem and Brüggemann put it, simply the possession and access to different resources at home, such as desk, computer, books, better and fashionable clothes, independently to the school infrastructure might influence as well school participation and attainment (2016, p. 19). Though statistically there was no association between the spending of remittances and finished school levels, mostly because almost all families received and spent money (and we did not ask about the amount received), remittances are more likely to have greater effect on the transition to higher secondary education, when there is greater tendency for older children to leave school to work.

Few of them graduate highschool, but this is I think a problem on national level, if you have 8^th^ classes you are finished, in the 9^th^ grade rarely see one [Roma pupil]. (…) because in many cases when a children reaches a certain age of 14 they have to work already, as daily-workers in agriculture and who knows where else. So this is it…the life conditions, but this is all up to the family. (…) They also go abroad for work…many of them…sometimes even the 8^th^ grade boys are missing from school, because they went to work [abroad]. (interview, school teacher, 2015, MigRom project, Baratca)

There are a spectrum of factors whose interplay can equally influence educational outcomes, remittance spending being just one of them. To this is closely connected the migration experience, that beside financial inputs, can contribute more with social remittances to changes in schooling practices. We compared those households that had migration experience with those who had not, to see whether there are significant differences.^11^

Table 8.3 shows the highest school level graduated by Roma persons that were born before 2005 comparing those who live in households where none of the family members had ever worked abroad with those where at least one family member had migration experience. There is a strong positive association (p ≤ 0.001) between migration experience and level of education, slightly stronger in Bighal than in Baratca. Those who live in households that had at least one family member working abroad seem to stay longer in school. In Bighal though we cannot separate (statistically speaking) the effect of migration from that of other influencing factors (like the effect of the community day-care, the educational projects implemented in the school, the remittances or the more tolerant and inclusive atmosphere in the village), but all in all, during our fieldworks the interviewees acknowledged that remittances (be that financial or social) contribute a lot in managing their relationship with the educational system: they can invest more in home milieu buying for example computers, can contribute more to the regular expenses required by the school, can buy more diversified food and more fashionable clothes for the children, and the list could end with greater ability to accommodate to strange situations with greater confidence. Sometimes there are contrasting interpretations on the reason why Roma children drop out from school at a younger age, consequently on the reason why there might be sensed improvements. When speaking about improvements in Baratca, one social worker said:

If you would have come few years ago, I would have said that there is a huge problem, because many of them are dropping-out, but right now I see that there are youngsters that graduate from school, even at the Bacalaureat level [12^th^ grade], but we have more with 8^th^ and 10^th^ classes now than earlier. But few years ago you could rarely see Roma children in the 8^th^ grade because they fell out somehow. Some of them started to work, they found something…so to say, and everything has an advantage also, because if there are no work opportunities, than children won’t go to work, but come to school. But in the last years, I have to say that I see that both the children and parents realized that it is better to go to school, because they would have a better future, if they have a certificate in their hands. (interview, social worker, 2015, MigRom project, Baratca)

**Table 8.3:** The highest graduated school level of Roma born before 2005 comparing households without and with migration experience (%)^a^

	BARATCA		BIGHAL	
	Without migration experience (%)	With migration experience (%)	Without migration experience (%)	With migration experience (%)
Without any education	25.6	13.1	6.7	3.1
Elementary school (I–IV grades)	30.8	28.7	25.8	12.8
Secondary school (V–VIII grades)	39.8	52.3	55.1	60.9
Highschool	3.8	5.8	11.8	22.3
University level (any type)	0	0	0.6	0.9
**Total persons**	**289**	**449**	**178**	**327**

Data source: MigRom project (RIRNM)

^a^Level = 0.000 with Cramer’s V = 0.175 for Baratca and Cramer’s V = 0.211 for Bighal. The variables were recoded and those who were born after 2005 were excluded from the analysis

Some of the families are very poor, but these children are really in a more difficult situation, but the school for example helped them a lot, because there are some programs, they have access to computers…I like that they come, every day, they want to come, the parents are coming, too, to ask about things, some of them can buy the necessary things…but some of them…so, there are possibilities, but you should make use of them…because there are parents, that don’t let the children to come…not many, but there are. (interview, Roma school mediator, 2014, MigRom project, Bighal)

In many interviews with non-Roma teachers, educators and representatives of local institutions it is mentioned that Roma parents do not recognize the importance of schooling, they do not help at home because they are not educated on their turn, they don’t have the right mentality. This attitude was documented in other schools as well. For example, Drown also found that while some teachers acknowledged the difficulties faced by Roma, they still consider that parents are not interested enough in education or children do not have the ability to succeed in school (Drown 2019). It is illustrative how the attitudes of some Roma parents are expressed—and even better reflected—in the complaints of a teenage girl from Bighal when asked about reasons of conflicts with her parents:

They told me to learn more, because I am not a good student… .She [my mother] told me, baby, you will be what you will be, just don’t be such a bad student like now, that you are the last in your class. Because she said that if we learn a bit better and I don’t know what, then we can do something with it. (interview, girl, 2009, Bighal)

Parents describe the perspective of a better future for their children:

I ask my children: what do you want? To go to the field with the hoe? No…don’t laugh [says to one of her children present at the discussion]. To have an easy job, a good employment…to have a better life than we had. I can’t complain myself…but if we had more money, it would be better…but we are satisfied with what we have now. School for them should be the priority. To be good students. Where is my boy? Here…he is 4 years old. When I ask him what does he want to become, he says policeman. …ah, that would be good…He can be, if he really wants. Because he is clever…he already speaks 3 languages almost…and he is just 4 [years old]… .Ah, their only job [childrens’] is to learn better.(…) (interview, woman, 2007, Bighal)

The discordance between the attitude of Roma parents regarding schooling and attitudes attributed to them by institutional representatives (speaking about failing children and failing parents) emphasizes the educational problem (Delamont 2014) and the lack of attention on inclusivity in school (Szalai and Schiff 2014).

## Conclusions and Ideas for Further Research

Exploring my experiences on the field and the data collected through qualitative and quantitative methods, I decided that is worthwhile to focus more on aspects that fall out of the immediate range of the strict framework of the school educational system, because in this way it can be shown that despite the inherent deficiencies of the system and the insufficient and inadequate policies, there might be initiatives, strategies or processes that challenge the constraints of well-established structures.

Most contributions to the literature on the education of Roma children highlights mostly discriminatory factors and contexts—be that intentional or not—along with well-intended affirmative actions and policies that contribute to the disadvantaged presence of Roma children in educational settings and processes (O’Nions 2007; Rostas 2012; Timmer 2017).^12^ Policy papers, recommendations in response address the absent or deficient needs and as a result interventions are designed, but these usually address immediate and short-termed needs or these are focused only on a restricted area ignoring the complexity of the problems.

The literature on the effects of migration on education presents a multifaceted situation depending on the focus of the analysis but also on the type of the community that was analyzed. For example, in some communities remittances raise school attendance, regardless of whether they have migration experience or not (Amuedo-Dorantes et al. 2010; Adams 2005; Zhunio et al. 2012), but sometimes factors that shape these outcomes are not conclusive (Viera 2018, p. 2; Lu 2014, p. 1095). There were case studies that found that migration and remittances had disruptive effects (Halpern-Manners 2011), despite their overall positive contribution to the well being of the household. Migration imposes an additional burden on the left-behind family members. Sometimes even contributes to the decreasing school attendance of school-age children because they had to take over the roles of the absent family member and to contribute to the household work. These may interfere with schooling even if financial remittances are available. So, in some cases, the investment of remittances does not have always a positive effect on the educational outcomes and social and economic mobility (Rao 2010, p. 142). Some other studies, on the contrary showed that financial remittances positively effect school participation especially in low-resourced areas and on poor and rural children, reducing drop-out rates and increases the probability of grade completion and/or continuation of schooling (Cox and Ureta 2003; Elrick 2008; Acharya and Leon-Gonzales 2014) in a number of ways (Zhunio et al. 2012, p. 4606), like increasing disposable income available for consumption, making possible to spend more on education and health, changes the composition of the household, so it might have both direct, but also indirect effects as well (building a bathroom, an additional room, investing in heating, clothing, and so on).

The panoply of factors, practices or mobilisable resources in improving educational system and process is large enough for one to get lost. In this chapter we identified two interventions or social processes that triggered perceptible change in the school participation of Roma youth, of which one is less entrenched in the existing literature on the education of Roma children. One is a project implemented with and by the local Roma community using extrinsic resources and the other one is the self- empowerment of the members of the local community who started to participate in the international migration in order to countervail the vanishing local job opportunities.

Both the Roma community center and the migration process contributed to the evolvement of mechanisms that worked as a kind of substitute to the lack of good school infrastructure, and deficient inclusive and minority sensitive education. The program of the community center contributed to the growth of sentiment of *familiarity* of both the children and parents with the pre-school program making easier the transition to the more inflexible school program. While the remittances and migration experience itself made it possible for the families *to conform* with greater confidence to the formal and informal expectations of school requirements.^13^ Otherwise, evidence showed that there is weak identification with the school as an institution and its norms (Voelkl 1997). Although it would be a hazardous hypothesis to conclude that these were the two factors that resulted in the improvement of school participation of the Roma children, their contribution to school participation—among other local processes or on the contrary the lack of it—is undeniable. Both processes contributed to relative improvements in the life conditions of families and these improvements could positively influence the school participation of poor and vulnerable children. Data showed that in the case of the older generation there was an association between living in segregated area of the village and school participation. Those who before 1990 lived in mixed neighborhoods had higher educational levels as compared to those who lived in segregated areas. While later in the case of the younger generations it seems that living in segregated areas (but still close to the village school) does not influence school attendance and attainment, but the effect of implemented projects and migration/ remittances grew significantly. Even in the case of Baratca where otherwise we found that the social distance between locals and the Roma is high and there are strong prejudices and stereotypes against Roma.

Despite the fact that the school educational system has some lacunas and it contributes to the reproduction of inequalities and even to the reproduction, reinforcement and interiorizing of prejudices, I argued that it is important to identify contexts and factors that although not immediately and directly but ultimately positively influence the school participation of Roma (minoritized, marginalized, vulnerable) children contributing in the longer term to the recognition and empowerment of Roma families.

## Footnotes

1 Bourdieu borrowed the model from the physicist James Clerk Maxwell, who imagined a demon which controls the door between two chambers of gas selecting the faster and slower molecules, thus maintaining the difference in the system, thereby entropy decreases and the second law of thermodynamics is violated.

2 Hereby I express my gratitude to the people of the villages that starting from 1999 they accepted my periodical presence in their lives, and to Cristina Raț and Fosztó László for the reinforcing and enlightening conversations. I remain deeply indebted to Rosa Drown for her endless support, numerous advices and her efforts to improve my text. I am also grateful to Judit Durst, Gyula Kozák, Zsuzsa Plainer and my colleagues from the DICE-ROMA workshops at the RIRNM for their insightful suggestions. All shortcomings weight solely on my shoulders.

3 In the MigRom project we surveyed 240 households in Baratca and 180 households in Bighal, comprising 1043, respectively 672 persons, out of which more than 80% live in compact Roma neighborhoods. The aim was to reach all self-identified Roma households from the villages (community census). More details on the socio-economic situation, demographic characteristics, migration experiences and patterns, generally more contextual data and methodology are described in Toma et al. (2018), Toma and Fosztó (2018a, b).

4 In Romania pre-school education is not compulsory yet, but a new Law of Education is in preparation that stipulates that until 2030 the latest, all levels of education, including preschool level, must be compulsory. https://senat.ro/Legis/Lista.aspx?cod=21292

5 In 2015 it was adopted a Strategy for Reducing of Early School Leaving in Romania, though it is too early to see whether it has been implemented effectively. https://www.edu.ro/strategia-privind-reducerea-p%C4%83r%C4%83sirii-timpurii-%C8%99colii-%C3%AEn-rom%C3%A2nia

6 Chi-square = 0.000 with Cramer’s V = 0.299. The variables were recoded and those who were not school-aged were excluded from the analysis.

7 They are Romani speakers, but fieldwork revealed that they use both Romanian and Hungarian in home and community context as well. Children thus have the opportunity to learn basic Romanian and Hungarian at home before being enrolled in school system. This is important, because language barriers affect academic achievement in schools (Feischmidt 2014).

8 Though there are more or less (less) rooted conventions on generation panels (e.g. X, the Millenials, Z and so on), we chose to ignore the global conventions, and to generate cohorts that better reflect local situations.

9 Chi-square = 0.000 with Cramer’s V = 0.276 for Bighal and Cramer’s V = 0.381 for Baratca. The variables were recoded and those who were not born in these periods were excluded from the anlaysis.

10 240 households in Baratca and 180 households in Bighal, comprising 1043, respectively 672 persons (Data source: MigRom Project).

11 We are aware that there might intervene the endogeneity effect as well (i.e. to separate the combined effect of migration and educational aspirations), but our data does not permit to separate these effects. Instead, we put the data in the light of the personal experiences of the participants themselves.

12 For a rich empirical and theoretical incursion in this topic you may see the Special Issue of Race, Ethnicity and Education 20(5) edited by Brüggemann and D’Arcy (2017). An overview of the dominant discourses in academic literature was published by Lauritzen and Nodeland (2018) in Educational Research Review.

13 Unfortunately, the word limits of this publication does not permit to enter into more details, but it is worth mentioning that Ogbu’s ‘ acting white’ theory does not have empirical support in this context: high-achieving students are likely to gain prestige, rather than lose it and being excluded (Ogbu 2004; Brüggemann 2014), with the careful note that it might contextual.
